# Efficacy and safety of Nirmatrelvir/Ritonavir for treating the Omicron variant of COVID-19

**DOI:** 10.3389/fmed.2023.1161193

**Published:** 2023-07-13

**Authors:** Chaochao Qiu, Zhengxing Wu, Xiaojing Liu, Qiang Zhang, Lianpeng Wu, Xinchun Ye, Jiajun Zhou, Jichan Shi, Xiangao Jiang

**Affiliations:** Department of Infection, Wenzhou Central Hospital, Dingli Clinical College of Wenzhou Medical University, Wenzhou, Zhejiang, China

**Keywords:** COVID-19, Omicron variant, Nirmatrelvir/Ritonavir, efficacy, adverse effect

## Abstract

**Objective:**

To evaluate the efficacy and safety of Nirmatrelvir/Ritonavir in the treatment of the Omicron variant of coronavirus disease 2019 (COVID-19).

**Methods:**

Data from 58 patients who were infected with the Omicron variant of COVID-19 were retrospectively collected. The patients were divided into two groups according to the treatment regimen they received. Patients in both groups were given Lianhua Qingwen capsules orally, three times/day, 6 g/time. The study group was given Nirmatrelvir 300 mg/Ritonavir 100 mg orally, q12h, for 5 days, and the control group was not given any antiviral drugs. The two groups were compared in terms of the change in computed tomography (CT) values of COVID-19 nucleic acid, the negative conversion time of COVID-19 RNA, hospitalization time, adverse drug reactions and COVID-19 nucleic acid re-positive tests.

**Results:**

The time to increase the CT values in the study group was faster than that in the control group, and the CT values in the study group were significantly larger than in the control group on days four and seven (*p* < 0.05); The negative conversion time in the study group was shorter than the control group (Z = –2.424, *p =* 0.015), and the hospitalization time was also shorter (Z = –2.603, *p* = 0.009). There were no statistically significant adverse drug reactions during hospitalization in both groups (χ^2^ = 2.747, *p* = 0.097). None of the study group tested re-positive for SARS-CoV-2 nucleic acid after discharge.

**Conclusion:**

The efficacy of Nirmatrelvir/Ritonavir in the treatment of the Omicron variant of COVID-19 was positive and had good tolerance in patients.

## 1. Introduction

Since the December 2019 outbreak of coronavirus disease 2019 (COVID-19), the epidemic has induced a global disease pandemic, with confirmed cases and cumulative deaths continuing to increase (1). COVID-19 has appeared in multiple variants, and the fifth generation variant of the virus is widely prevalent worldwide, which the World Health Organization named Omicron (2). Due to the emergence of the Omicron variant, the protective effect of vaccines may be weakened (3). Therefore, the discovery of effective and safe antiviral drugs is key to reducing the global disease burden.

The emergence of Nirmatrelvir/Ritonavir (Paxlovid) has brought new hope for the treatment of COVID-19, showing an effective inhibitory effect on Omicron (4, 5). Paxlovid is approved by the US Food and Drug Administration for the treatment of patients over 12 years old with mild to moderate symptoms and a high risk of disease progression. It has been approved by the State Food and Drug Administration of China for the treatment of adults over 18 years of age with patients with mild to novel coronavirus who have progressed to severe high-risk factors to reduce hospitalization and mortality risk (6). On 11 February 2022, according to the relevant provisions of the Drug Administration Law, the State Food and Drug Administration of China approved the import registration of the COVID-19 therapeutic drug combination package (Paxlovid) that was developed by Pfizer, which was used to treat adult patients with mild to moderate COVID-19 with high-risk factors for progression to severe symptoms. A retrospective study on 58 patients with coronavirus and pneumonia who were infected with the homologous Omicron variant has been designed to discuss the efficacy and safety of Nirmatrelvir/Ritonavir for treating the Omicron variant. The data were compared and analyzed.

## 2. Materials and methods

### 2.1. General materials

A total of 58 patients with COVID-19 who were isolated and treated in Wenzhou Central Hospital in August 2022 were retrospectively collected. They were diagnosed with the homologous Omicron BA.2.76 variant infection by Wenzhou Center for Disease Control and Prevention. They were then divided into two groups according to different treatment regimens. Among them, 15 patients in the study group were treated with Nirmatrelvir/Ritonavir, and 43 patients in the control group received routine treatment. All patients were informed and agreed to participate in the research, and the Hospital Medical Ethics Committee approved the study protocol.

### 2.2. Selection criteria

#### 2.2.1. Inclusion criteria

① Confirmation of COVID-19 infection within 24 h and positive nasopharyngeal swab for COVID-19 nucleic acid RNA; ② Age ≥ 12 years and weight ≥ 40 kg; ③ Subjects of fertility must agree to use highly effective contraceptive methods; ④ According to the Chinese ‘new coronavirus infection diagnosis and treatment plan (trial version 10),’ patients who were diagnosed as mild, moderate, severe and critical types who required hospitalization were included.

#### 2.2.2. Exclusion criteria

① Previous history of COVID-19 treatment; ② A known history of active liver disease; ③ Patients on renal dialysis or who had moderate to severe impaired renal function; ④ Patients with known human immunodeficiency virus (HIV) infection; ⑤ Suspected or confirmed concurrent active systemic infections other than COVID-19 infection. ⑥ Allergy or other contraindication to any component of the study intervention; ⑦ Any drug or substance that is currently or expected to be used that has a high reliance on CYP3A4 enzyme clearance or strong CYP3A4 enzyme inducers; ⑧ Pregnant or breastfeeding women (7, 8).

#### 2.2.3. Discharge criteria

**Figure 1:**
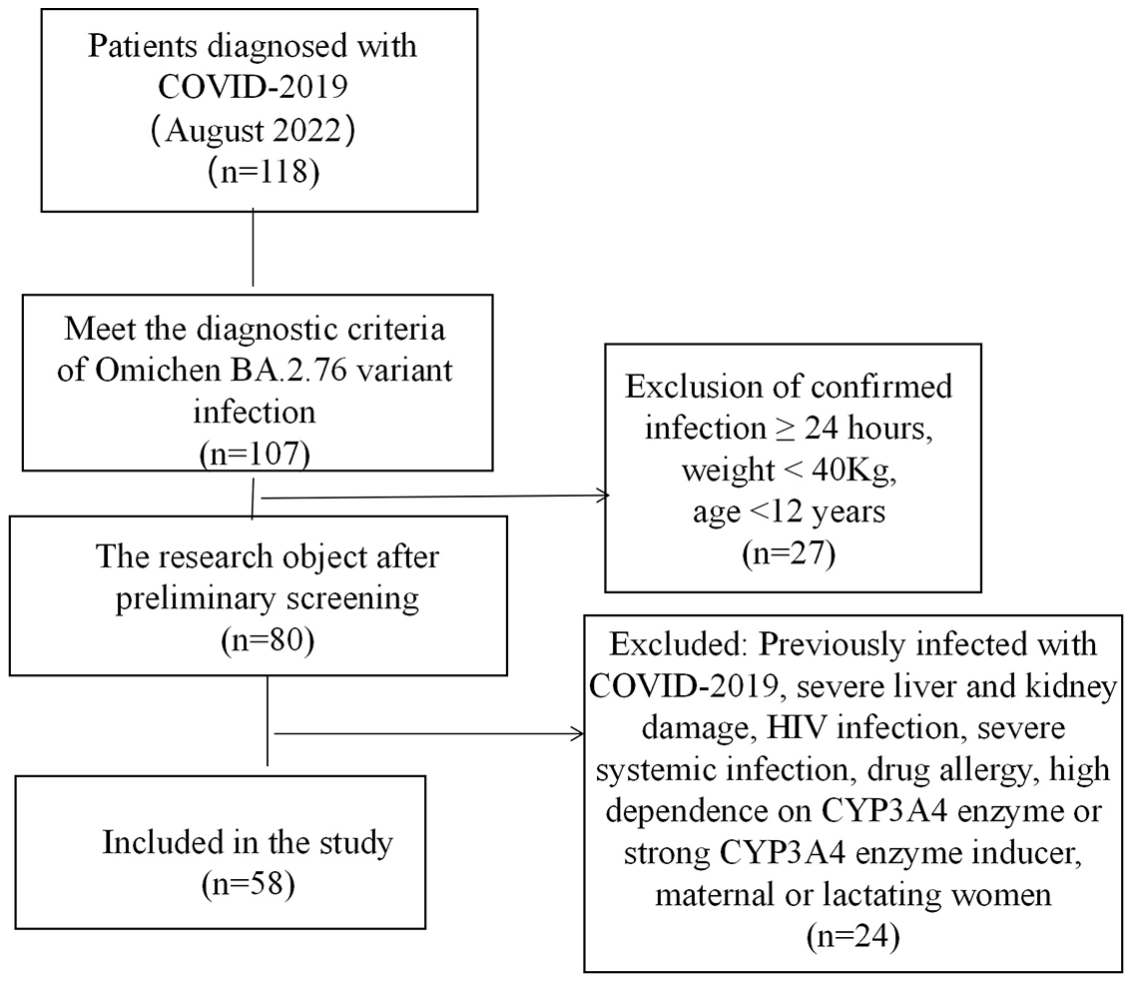
Screening process of research subjects.

(1) Temperature back to normal more than 3 days; (2) Significant improvement in respiratory symptoms; (3) Lung imaging showed a marked improvement in acute exudative lesions; (4) CT values of N gene and open reading frame (ORF) gene for two consecutive COVID-19 nucleic acid tests were ≥ 35 [fluorescent quantitative polymerase chain reaction (PCR) techniques with a threshold limit value of 40 and a sampling interval of at least 24 h], or COVID-19 nucleic acid test negative for two consecutive (fluorescent quantitative PCR techniques with a threshold limit value below 40 and a sampling interval of at least 24 h) (9). Those who were satisfied with the above requirement could be discharged. The screening process of the research object is shown in Figure 1.

### 2.3. Methods

Patients in both groups were given Lianhua Qingwen Capsule orally, three times/day, 6 g/time, oral antipyretic (ibuprofen suspension 10 mL) and symptomatic supportive treatment for a body temperature of >38.5°C. The study group was given Nirmatrelvir 300 mg/Ritonavir 100 mg orally, q12h, for 5 days, and the control group was not given any antiviral drugs.

### 2.4. Outcome measures

① Patients in both groups underwent nasal swab COVID-19 nucleic acid tests on days four, seven, nine and 11 of treatment, and their computed tomography (CT) values were recorded (CT values of 40 for negative results). The differences in the change of CT values of COVID-19 nucleic acid between the two groups of patients were compared; ② The first negative conversion (or CT value of ≥35) time of coronavirus nucleic acid between the two groups was compared; ③ The difference in the hospitalization time between the two groups was strictly compared according to the discharge criteria; ④ The adverse drug reactions during hospitalization between the two groups were compared; ⑤ After discharge, patients in both groups continued to be isolated at the isolation point for 7 days and underwent COVID-19 nucleic acid tests daily to compare the COVID-19 nucleic acid re-positive tests.

### 2.5. Statistical methods

Data were processed using SPSS version 19.0 statistics. The measurement data were statistically described using mean ± standard deviation, and the count data were statistically described by the number of cases or percentage. The measurement data were tested for normality and homogeneity of variance before the groups were compared. If the normality and homogeneity of variance were satisfied, the two-sample independent *t-*test was used. If not satisfied, the non-parametric statistical test (Mann–Whitney method) was used, and the χ2 test was used for the comparison between groups. A result of *p* < 0.05 was considered a statistically significant difference.

## 3. Results

### 3.1. Baseline characteristics of research objects

In the study group, there were seven males and eight females in 15 cases with a mean age of 31.7 ± 8.6 years (ranging from 18 to 50 years). Clinical typing was light; 14 cases were vaccinated, and one case was unvaccinated. One of these cases had a history of ‘systemic lupus erythematosus,’ and the rest had no underlying diseases. In the control groups, there were 19 males and 24 females in a total of 43 cases with a mean age of 36.7 ± 12.3 years (ranging from 13 to 57 years). Clinical typing was mild type infected individuals in eight cases and medium-heavy type in 35 cases; 42 cases had been vaccinated, and one case had not been vaccinated. Two of these cases had a history of ‘hypertension,’ and one had a history of ‘diabetes.’ The rest had no underlying diseases. The basic data of the two groups were balanced and comparable (*p* > 0.05), as shown in Table 1.

**Table 1:** Comparison of basic materials between two groups of patients.

Features	Research group	Control group	*t/χ^2^/Z*	*P*-value
Age ( $\bar{x}$ ±s, years)	31.7 ± 8.6	36.7 ± 12.3	1.451	0.152
Gender			0.079	0.778
Male [cases(%)]	7(46.7%)	19(44.2%)	-	
Female [cases(%)]	8(53.3%)	24(55.8%)	-	
Admission of nucleic acid CT values
N gene[M,(IQR)]	20.2(6.9)	19.7(5.6)	−0.186	0.852
ORF1a/b gene[M,(IQR)]	21.7(7.3)	20.2(5.1)	−0.595	0.552
Underlying disease [cases (%)]	1(6.7%)	3(7.0%)	0.002	0.967
The type of vaccination [cases (%)]			1.260	0.262
Inactivated vaccine	10(71.4%)	36(85.7%)		
Recombinant protein vaccine/Adenovirus vector vaccine	4(28.6%)	6(14.3%)		
Number of vaccinations			3.675	0.159
1 injection	3	2		
2 injection	4	10		
3 injection	8	31		
Laboratory tests			3.058	0.151
White-cell Count [×10^9^/L]	7.4 ± 2.4	7.7 ± 2.2	0.407	0.686
CRP[mg/L,M,(IQR)]	2.2(4.6)	2.1(6.9)	−0.595	0.552
Clinical typing [cases (%)]			3.237	0.072
Mild type	0(0%)	8(18.6%)		
Medium-heavy type	15(100%)	35(81.4%)		

CT, computed tomography; M, median; IQR, interquartile range; ORF, open reading frame. T, two-sample independent *t*-test. χ^2^, χ^2^ test. Z, non-parametric statistical test (Mann–Whitney method).

### 3.2. Change of COVID-19 nucleic acid computed tomography values

**Figure 2:**
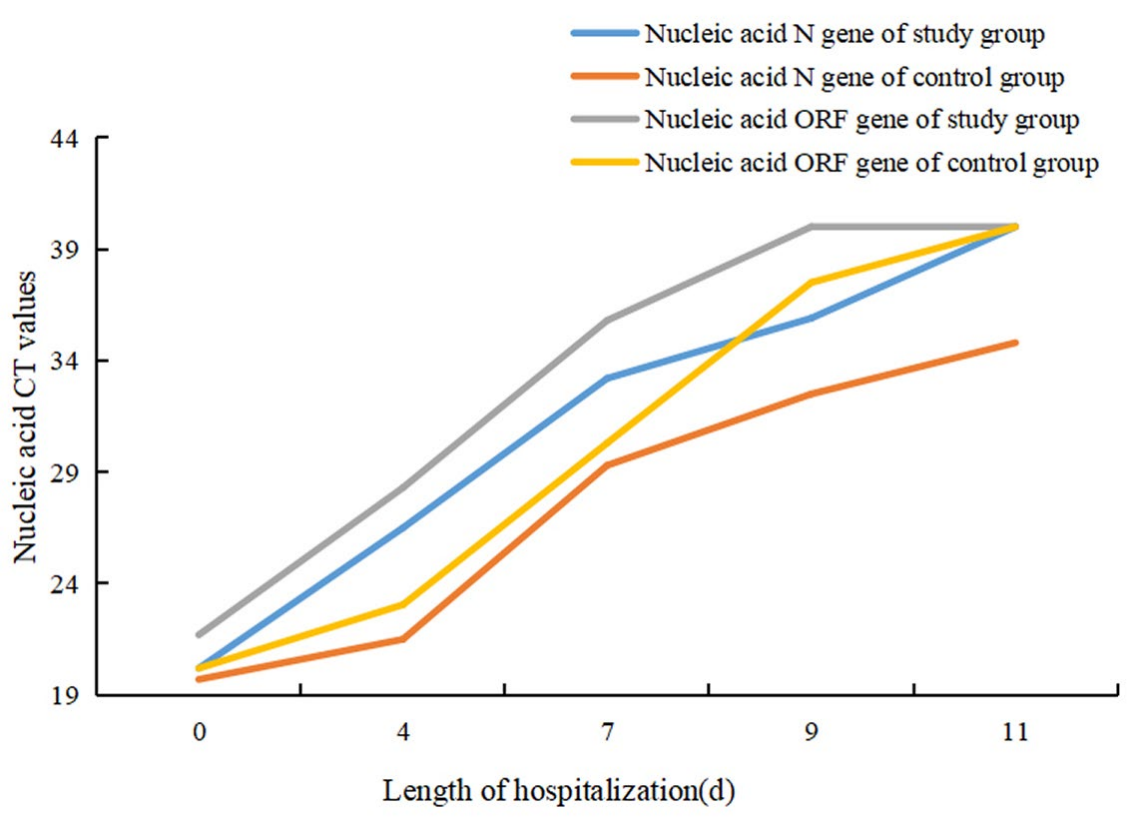
Change curve of nucleic acid CT values in two groups of patients.

Patients in both groups underwent nasal swab COVID-19 nucleic acid tests on days four, seven, nine and 11 of treatment. The CT values were recorded (N gene and ORF gene, CT values of 40 for negative results). The COVID-19 nucleic acid CT values in the study group were significantly greater than those in the control group on days four and seven, and the difference was statistically significant (*p* < 0.05). The CT values of N gene on the ninth and 11th days of the study group were higher than that of the control group, and the difference was statistically significant (*p* < 0.05), as shown in Table 2. The baseline of the nucleic acid CT values was similar between the two groups at the time of admission, and the COVID-19 nucleic acid CT values in the study group increased more rapidly than those in the control group during the treatment period, as shown in Figure 2.

**Table 2:** Change of nucleic acid CT values in two groups of patients.

Features	Study group	Control group	*Z/t*	*P*-value
Day 4 nucleic acid CT values
N gene[M,(IQR)]	26.5(6.8)	21.5(5.9)	−4.280	<0.001
ORF1a/b gene	29.9 ± 4.8	23.0 ± 4.0	5.364	<0.001
Day 7 nucleic acid CT values				
N gene[M,(IQR)]	33.2(10.1)	29.3(6.2)	−3.280	0.001
ORF1a/b gene[M,(IQR)]	35.8(7.0)	30.3(6.6)	−3.467	0.001
Day 9 nucleic acid CT values
N gene[M,(IQR)]	35.9(5.7)	32.5(10.1)	−2.253	0.024
ORF1a/b gene[M,(IQR)]	40.0(2.7)	37.5(8.4)	−1.614	0.107
Day 11 nucleic acid CT values
N gene[M,(IQR)]	40.0(1.0)	34.8(7.9)	−2.013	0.044
ORF1a/b gene[M,(IQR)]	40.0(0.0)	40.0(5.3)	−0.311	0.755

CT, computed tomography; M, median; IQR, interquartile range; ORF, open reading frame. T, two-sample independent *t*-test. Z, non-parametric statistical test (Mann–Whitney method).

### 3.3. The first negative conversion time of COVID-19 nucleic acid

The first negative conversion time of COVID-19 nucleic acid in the two groups was counted. The time of negative conversion in the study group ranged from four to 21 days, and the median of the first negative conversion time was 9. The time of negative conversion in the control group range from six to 22 days, and the median of the first negative conversion time was 14. The difference was statistically significant (*Z* = –2.424, *p* = 0.015). See Table 3.

**Table 3:** Comparison of relevant indicators between two groups of patients.

Observed indicators	Study group	Control group	*t/χ^2^*	*P*-value
The negative conversion time of nucleic acid [day, M,(IQR)]	9.0(6.0)	14.0(7.0)	−2.424	0.015
The length of hospitalization [day, M,(IQR)]	13.0(6.0)	18.0(6.0)	−2.603	0.009
Adverse drug reactions [cases(%)]	2(13.3%)	1(2.3%)	2.747	0.097
Nucleic acid re-positive [cases(%)]	0(0%)	7(16.3%)	2.777	0.096

M, median; IQR, interquartile range. T, two-sample independent t test. χ^2^, χ^2^ test.

### 3.4. Length of hospitalization

According to the discharge criteria, the two groups’ hospitalization lengths were counted. The length of hospitalization of the study group was 13 days (ranging from eight to 25 days). The control group’s hospitalization length was 18 days (ranging from 10 to 27 days). The difference was statistically significant (Z = –2.603, *p* = 0.009). See Table 3.

### 3.5. Adverse drug reactions

During hospitalization, the blood routine and biochemical indexes from the patients in both groups were reviewed regularly, and adverse drug reactions were counted. There were two cases (13.3%) of adverse drug reactions in the study group: allergic reaction to drugs and transaminase elevation [glutamic pyruvic transaminase (ALT) elevation <2 times Upper limit of liver function reference value (ULN)] respectively, which improved after symptomatic treatment, and none of the antiviral drugs were discontinued during this period. There was one case (2.3%) of transaminase elevation (ALT elevation <2 times ULN), which improved after symptomatic treatment. The data difference was statistically insignificant (χ^2^ = 2.747, *p* = 0.097). See Table 3.

### 3.6. COVID-19 nucleic acid re-positive

After discharge, patients in both groups continued to be isolated at isolation points for 7 days and underwent COVID-19 nucleic acid tests daily. There were no re-positive patients in the study group, whereas seven patients tested re-positive in the control group. There was no statistically significant difference in the re-positive group (χ^2^ = 2.777, *p* = 0.096). See Figure 2.

## 4. Discussion

At present, the Omicron variant has become a major strain of the COVID-19 global pandemic, and its extreme infectivity and immune evasion (10, 11) make prevention and control of the epidemic particularly difficult. Due to the many mutations of Omicron, the Omicron is able to escape the immune system’s defenses and coronavirus disease 2019 vaccines are less effective against the Omicron variant (12). some COVID-19 antibody drugs appear to be ineffective against this strain (13, 14), and the National Institute of Health believes that Nirmatrelvir/Ritonavir may be in the minority of drugs that are still effective against the Omicron variant (15, 16). Nirmatrelvir/Ritonavir is a combination package of Nirmatrelvir 150 mg and Ritonavir 100 mg and is applicable for treating mild to moderate coronavirus disease (COVID-19) in adults and pediatric patients (12 years of age and older weighing at least 40 kgs) and the patient population at high risk for progression to severe COVID-19. Nirmatrelvir works by inhibiting the main protease (Mpro) of SARS-Cov-2, also known as 3C protease (17), which is the major protease that shears and processes RNA in the COVID-19 self-coding, and its deactivation inhibits viral replication (11). Ritonavir, a HIV-1 protease inhibitor already in clinical use, is not active against SARS-CoV-2 Mpro. It increases Nirmatrelvir activity in plasma at higher concentrations by inhibiting CYP3A mediated metabolism (7, 18).

In this study, through a comparative analysis of 58 patients infected with the homologous Omicron variant of COVID-19, it was found that the time to increase the CT values of COVID-19 nucleic acid was faster in the study group than in the control group. Comparing the change curves of COVID-19 nucleic acid CT values in the two groups of patients showed that the curve in the study group shifted to the left compared with the control group. This indicates that the administration of Nirmatrelvir/Ritonavir can inhibit the replication of COVID-19 and shorten the detoxification time. By comparing the time of nucleic acid negative conversion, the median of the first negative conversion time was 9 (6) in the study group and 14 (7) days in the control group. The time in the study group was shorter than in the control group, indicating that the administration of Nirmatrelvir/Ritonavir can shorten the time of nucleic acid negative conversion in patients. When comparing the hospitalization lengths, it was found that the average hospitalization length was 13 (6) days in the study group and 18 (6) days in the control group. The length of hospitalization was shorter in the study group compared with the control group, indicating that the administration of Nirmatrelvir/Ritonavir can shorten the length of hospitalization in patients, reduce medical resources and ease the medical burden. There are some reports (19) that patients with COVID-19 who were treated with Nirmatrelvir/Ritonavir experienced rebound and nucleic acid re-positive tests. Patients of the study group the stable disease during the treatment period in the study, and there were no rebound cases during the isolation period after the patients were discharged from the hospital. This study does not support the reported phenomenon of rebound and nucleic acid re-positive tests after treatment with Nirmatrelvir/Ritonavir, and further studies are needed to provide relevant evidence. There was no significant difference between the two groups in this study, indicating that Nirmatrelvir/Ritonavir has good overall patient tolerance. This is consistent with the research results by Liu Xiaolin et al. (20), which found that two cases (13.3%) of adverse drug reactions occurred in the study group, including drug allergy and elevated transaminase. Nirmatrelvir/Ritonavir has a high affinity for cytochrome P450 (CYP) subtypes, especially for CYP3A4 and CYP2D6 (5). However, this study did not genotype the patients before and after treatment, so it is not possible to determine whether the adverse drug reactions in patients were due to the interaction between the genotypes and drugs or due to the adverse reactions of the patients’ own underlying diseases.

Limitations: First, due to the research background, the sample size was a problem. However, with the current changes in the epidemic situation, more subjects will be added to the clinical trials of new coronavirus pneumonia drug therapy. Therefore, a multi-center randomized double-blind trial can be carried out in the future to verify the safety and efficacy of Nirmatrelvir/Ritonavir tablets. Second, the rationality of drug use had not been evaluated from the aspects of drug indications, use and dosage, timing of administration, course of treatment, combination of drugs, and interaction between the genotypes and drugs. Therefore, more real-world data should be applied to analyze the rationality of Nirmatrelvir/Ritonavir tablets in future research. Third, no positive control was established in this study. As a result, the Nirmatrelvir/Ritonavir treatment was not compared with other therapies in the study. Finally, this study has not yet considered the correlation between the level of cellular and humoral immunity and clinical symptoms before and after treatment from the perspective of immunity, so the level of immunity should be considered in future studies.

In summary, Nirmatrelvir/Ritonavir tablets are effective in the treatment of the COVID-19 Omicron variant, and the overall tolerance of patients is good. It plays an important guiding role in the treatment of novel coronavirus pneumonia Omicron variants in the future, which can reduce the pain and disease burden of patients.

## Data availability statement

The raw data supporting the conclusions of this article will be made available by the authors, without undue reservation.

## Ethics statement

The studies involving human participants were reviewed and approved by Wenzhou Central Hospital. Written informed consent for participation was not required for this study in accordance with the national legislation and the institutional requirements.

## Author contributions

CQ, XJ, and JS: conception and design of the research. ZW, XL, QZ, and LW: acquisition of data. XY and JZ: analysis and interpretation of the data. ZW and XL: statistical analysis. CQ, ZW, and XL: writing of the manuscript. JS and XJ: critical revision of the manuscript for intellectual content. All authors contributed to the article and approved the submitted version.

## Conflict of interest

The authors declare that the research was conducted in the absence of any commercial or financial relationships that could be construed as a potential conflict of interest.

## Publisher’s note

All claims expressed in this article are solely those of the authors and do not necessarily represent those of their affiliated organizations, or those of the publisher, the editors and the reviewers. Any product that may be evaluated in this article, or claim that may be made by its manufacturer, is not guaranteed or endorsed by the publisher.
